# Design of Multifaceted Antioxidants: Shifting towards Anti-Inflammatory and Antihyperlipidemic Activity

**DOI:** 10.3390/molecules26164928

**Published:** 2021-08-14

**Authors:** Ariadni Tzara, George Lambrinidis, Angeliki Kourounakis

**Affiliations:** Division of Pharmaceutical Chemistry, Department of Pharmacy, School of Health Sciences, National & Kapodistrian University of Athens, 15771 Athens, Greece; atzara@pharm.uoa.gr (A.T.); lambrinidis@pharm.uoa.gr (G.L.)

**Keywords:** antioxidant, anti-inflammatory, multitarget, NSAID, LOX, in vitro, in vivo, in silico

## Abstract

Oxidative stress and inflammation are two conditions that coexist in many multifactorial diseases such as atherosclerosis and neurodegeneration. Thus, the design of multifunctional compounds that can concurrently tackle two or more therapeutic targets is an appealing approach. In this study, the basic NSAID structure was fused with the antioxidant moieties 3,5-di-tert-butyl-4-hydroxybenzoic acid (BHB), its reduced alcohol 3,5-di-tert-butyl- 4-hydroxybenzyl alcohol (BHBA), or 6-hydroxy-2,5,7,8-tetramethylchromane-2-carboxylic acid (Trolox), a hydrophilic analogue of α-tocopherol. Machine learning algorithms were utilized to validate the potential dual effect (anti-inflammatory and antioxidant) of the designed analogues. Derivatives **1**–**17** were synthesized by known esterification methods, with good to excellent yields, and were pharmacologically evaluated both in vitro and in vivo for their antioxidant and anti-inflammatory activity, whereas selected compounds were also tested in an in vivo hyperlipidemia protocol. Furthermore, the activity/binding affinity of the new compounds for lipoxygenase-3 (LOX-3) was studied not only in vitro but also via molecular docking simulations. Experimental results demonstrated that the antioxidant and anti-inflammatory activities of the new fused molecules were increased compared to the parent molecules, while molecular docking simulations validated the improved activity and revealed the binding mode of the most potent inhibitors. The purpose of their design was justified by providing a potentially safer and more efficient therapeutic approach for multifactorial diseases.

## 1. Introduction

Non-steroidal anti-inflammatory drugs are the most useful and commonly prescribed treatment for inflammatory conditions. Nevertheless, their wide use has been linked to many adverse effects, either minor (e.g., gastrointestinal irritation) or major (e.g., increased risk for cardiovascular disease) [1,2]. An interesting approach to overcoming these risks includes the design of dual cyclooxygenase (COX) and lipoxygenase (LOX) inhibitors, so as to inhibit the biosynthesis of both prostaglandins and leukotrienes [3]. The implication of reactive oxygen species in inflammatory conditions is well proven [4], mainly via the proinflammatory properties of the generated superoxide anion. The latter is involved in the deterioration of a plethora of inflammatory conditions. Moreover, radical intermediates are involved in the catalytic activity of COX, thus providing a possible way for radical scavengers to interfere with the enzyme’s activity [3]. 

**Figure 1:**
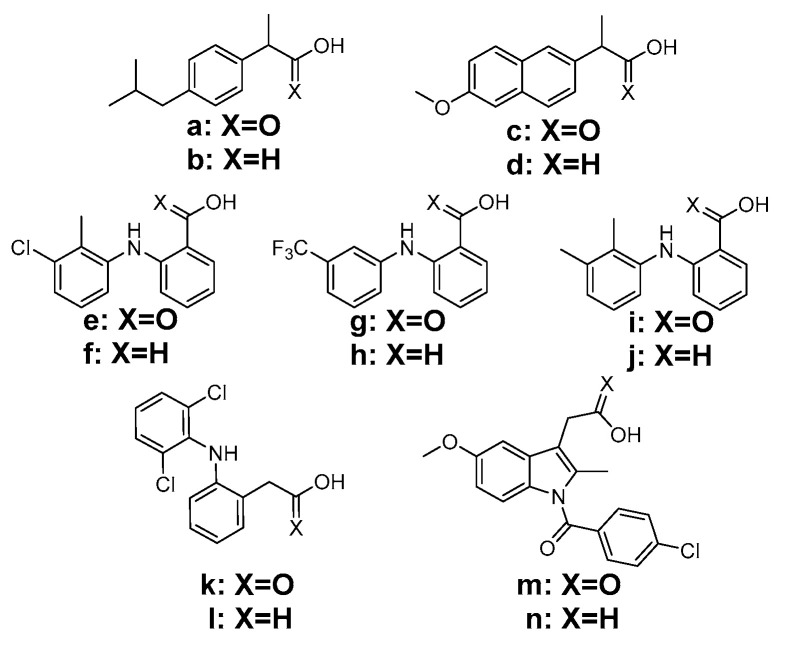
Structures of the used NSAIDs (acids) and their corresponding alcohols (indicated with an “-ol” suffix): (**a**) Ibuprofen; (**b**) Ibuprofenol; (**c**) Naproxen; (**d**) Naproxenol; (**e**) Tolfenamic acid; (**f**) Tolfenamol; (**g**) Flufenamic acid; (**h**) Flufenamol; (**i**) Mefenamic acid; (**j**) Mefenamol; (**k**) Diclofenac; (**l**) Diclofenol; (**m**) Indomethacin; and (**n**) Indomethacinol.

In these terms, the combination of a NSAID with a known antioxidant moiety in one structure may be an appealing approach towards the improvement of its therapeutic profile; this strategy has been researched via the conjugation of known NSAIDs with antioxidant moieties, mainly via amidation [5,6,7,8,9,10,11,12]. In this way, the derived compounds not only acquire antioxidant activity, but also show a much lower gastrointestinal toxicity, which is one of the main side effects of NSAIDs, especially upon chronic use. The carboxylic group of the parent NSAIDs is not absolutely necessary for the anti-inflammatory activity, making it a suitable and convenient functionalization group for the reduction in its acidic character and hence a much safer profile [5,6,7,8,9,10]. In this study, the NSAIDs or their corresponding alcohols, as presented in Figure 1, were fused with 3,5-di-tert-butyl-4-hydroxybenzoic acid (BHB), or its reduced alcohol 3,5-di-tert-butyl-4-hydroxybenzyl alcohol (BHBA), known for its LOX and COX inhibitory and interleukin-1-suppressing activity [13], as well as 6-hydroxy-2,5,7,8-tetramethylchromane-2-carboxylic acid (Trolox), a hydrophilic analogue of α-tocopherol with a more potent antioxidant efficacy [14].

## 2. Results and Discussion

### 2.1. Ligand-Based Activity Validation of Designed Compounds Using Machine Learning Algorithms

The designed compounds belong to our first attempt to explore the chemical space of such antioxidant-modified NSAIDs. Thus, we constructed a ligand-based filter using machine learning algorithms to strengthen our hypothesis prior to synthesis. Knowledge from already in vitro tested compounds can establish an accurate filter algorithm to separate compounds with high, moderate or low affinity. We constructed a classification protocol to check whether the compounds designed could be LOX and/or DPPH inhibitors as an indication of in vitro anti-inflammatory and/or antioxidant activity, respectively. Starting from the ChEMBL database [15], we selected molecules with an IC_50_ lower than 1000 nM against LOX-3 (Class 1) or DPPH (Class 2). We created an additional decoy set (Class 3) with 500 compounds for each target with IC_50_ greater than 1000 nM (see details in Materials and Methods). For all selected compounds, we calculated 277 molecular descriptors using the Schrodinger Suite 2018. MATLAB v2018b Classification Learner was utilized to select the most accurate machine learning algorithm for discrimination. The Cubic Support Vector Machine gave 97.7% prediction accuracy, as shown in the confusion matrix (see Supplementary Material, Figure S1).

The compounds designed for synthesis passed through the workflow. Compounds **4**, **7**, **8**, **9**, **10**, and **13** were classified as potent LOX-3 inhibitors; and **13**, **14**, **15**, and **16** as potent DPPH inhibitors; while the rest were potentially inactive. These classification protocol results encouraged us to proceed with the synthesis of all newly designed compounds since their unique and combined scaffolds could retrieve new lead compounds with dual activity.

### 2.2. Chemical Synthesis

**Figure 2:**
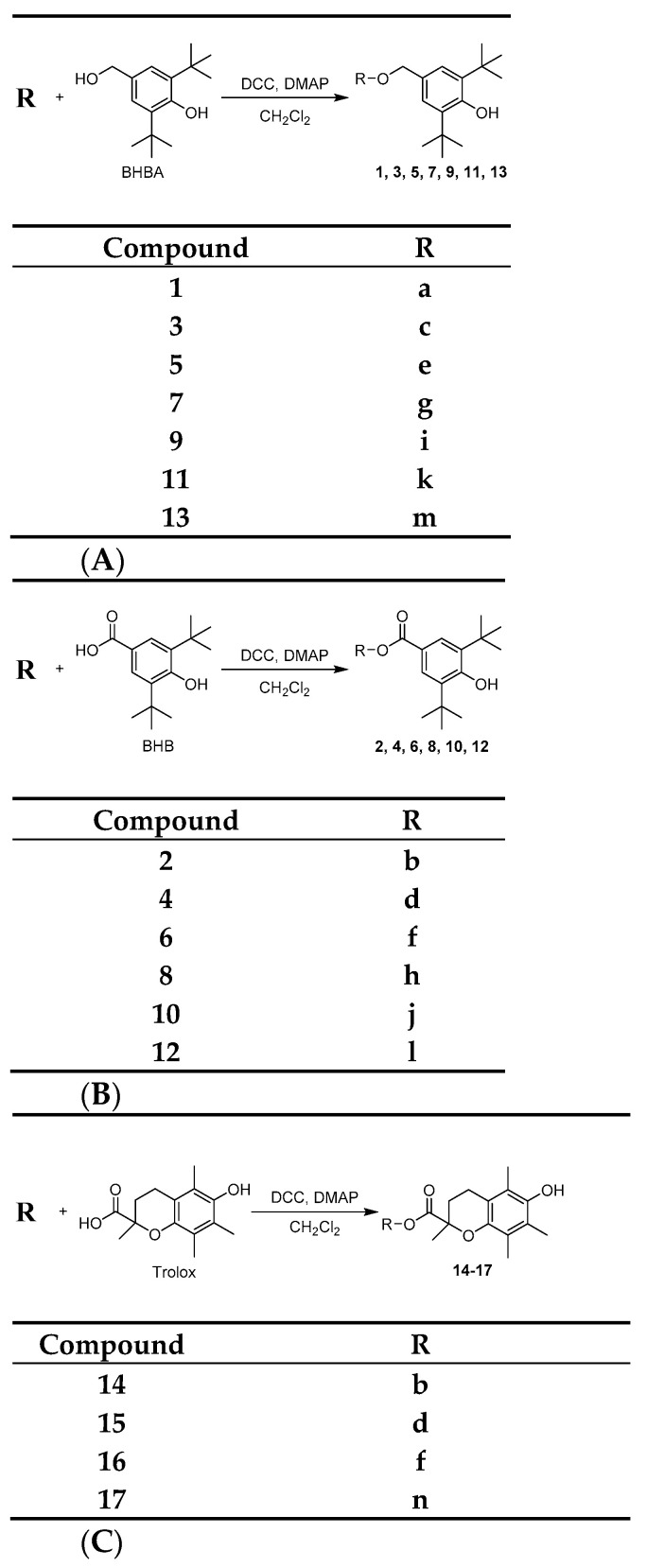
(**A**) Synthesis of derivatives **1**, **3**, **5**, **7**, **9**, **11,** and **13**; (**B**) synthesis of derivatives **2**, **4**, **6**, **8**, **10** and **12**; and (**C**) synthesis of derivatives **14**–**17**. (*DCC: N,N’-dicyclohexylcarbodiimide, DMAP: 4-dimethylaminopyridine*).

Derivatives **1**–**17** were synthesized by the esterification of the respective NSAID or the corresponding alcohol of NSAID (Figure 1, **a**–**n**) with BHBA, BHB or Trolox (as shown in Figure 2), using DCC and DMAP, from 0 °C to room temperature and with moderate to excellent yields (10–94%).

### 2.3. Evaluation of Pharmacological Activity

**Figure 3:**
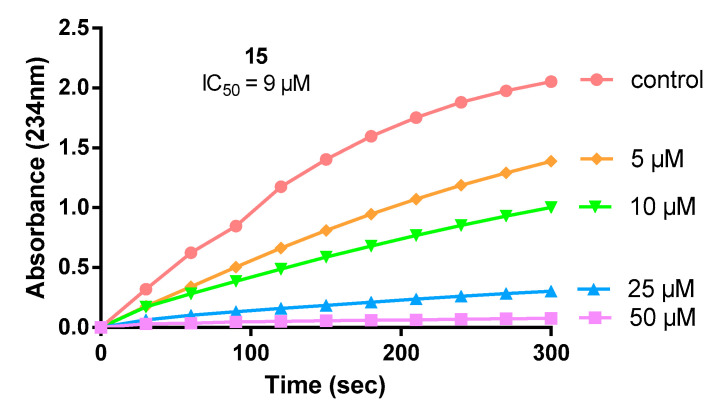
Representative graph showing the time-dependent inhibition of LOX-3 by different concentrations of derivative **15**.

The anti-inflammatory effect of the synthesized compounds was initially evaluated in vitro, via their ability to inhibit soybean lipoxygenase (LOX-3). The active site of this enzyme bears a great structural and sequence similarity to that of the mammalian enzyme 5-LOX, rendering soybean lipoxygenase a useful model for the evaluation of in vitro anti-inflammatory properties of novel compounds [16]. The effect of the synthesized compounds, as well as their parent molecules, on LOX-3 was evaluated and the results for the most potent are shown in Table 1 as IC_50_ values after 5 min of incubation. A representative graph of the time-dependent inhibition of the most active compound **15** is depicted in Figure 3. It is notable that compound **15** was not predicted as a potent LOX-3 inhibitor in our initial machine learning classification, due to the fact that our novel combined scaffold was absent in the ChEMBL database. The parent NSAIDs and antioxidants BHB, BHBA and Trolox have little or no inhibitory activity, except for Naproxen (**c**), and its reduced analog (alcohol) **d**, which exhibits IC_50_ values of 25 and 29 μM, respectively. The little or no inhibition (IC_50_ > 200 μM) of LOX-3 by most other parent NSAID molecules (**a**–**n**), as also observed in the literature [17,18,19], supports the fact that the incorporation of the two pharmacophores in one structure leads to an improved interaction with the enzyme.

In vivo anti-inflammatory activity of the synthesized compounds was evaluated as mouse paw edema reduction. Paw edema was induced by subcutaneous injection of a 2% carrageenan suspension in the right paw of mice, the left serving as the control [20]. The activity of the tested compounds (at 0.30 mmol/kg of body weight, *i.p.*), expressed as % reduction in paw edema 3.5 h after carrageenan injection, is shown in Table 2. The tested antioxidant-modified NSAID derivatives showed an improved anti-inflammatory profile compared to the parent NSAIDs. Compounds **7** and **8** (antioxidant derivatives of flufenamic acid) especially demonstrated a 2–2.5-fold improvement in anti-inflammatory activity, compared to the parent flufenamic acid (**g**) or flufenamol (**h**). Moreover, compounds **9** and **10** (antioxidant derivatives of mefenamic acid) caused a 1.5–2-fold improvement in edema reduction compared to that of the parent mefenamic acid (**i**).

The antioxidant activity of compounds **1**–**17** was evaluated via their ability to scavenge the free radical DPPH. No significant activity was found for most derivatives, except for reference compounds BHB (IC_50_ = 31 μM) and BHBA (IC_50_ = 160 μM), as well as the parent compound Trolox (IC_50_ = 33 μM) and its derivatives **14**, **15**, **16**, and **17**, which had IC_50_ values of 34, 147, 47, and 76 μM, respectively. The antioxidant (radical scavenging) profile of these compounds (**14**–**17**) was probably due to the moiety of Trolox that was incorporated in their structure, since the parent NSAIDs do not show any such activity. 

The antioxidant effect of the new compounds, as well as their parent compounds was also evaluated by the protection offered against rat/mouse microsomal lipid peroxidation. Results were expressed as IC_50_ values after 45 min of incubation [21,22,23,24,25,26]. Parent NSAIDs and their reduced derivatives alone had no significant activity against lipid peroxidation, with the exception of tolfenamic and mefenamic acid, having IC_50_ values of 250 and 130 μM, respectively [5]. On the other hand, reference compounds BHBA and Trolox had significant antioxidant activity, bearing an IC_50_ of 10 μΜ and 25 μΜ, respectively, whereas BHB had a greater IC_50_ value (257 μM). The incorporation of the two most active moieties of BHBA and Trolox in derivatives **9** and **14**–**17** led to a 25-fold increase in activity; compound **9** demonstrated a potent effect (IC_50_ = 5μΜ), compared to the aforementioned parent mefenamic acid (**i**). Compounds **14**–**17** showed a complete inhibition of microsomal lipid peroxidation at 50 μΜ, with an estimated IC_50_ value of ca. 10 μM.

**Figure 4:**
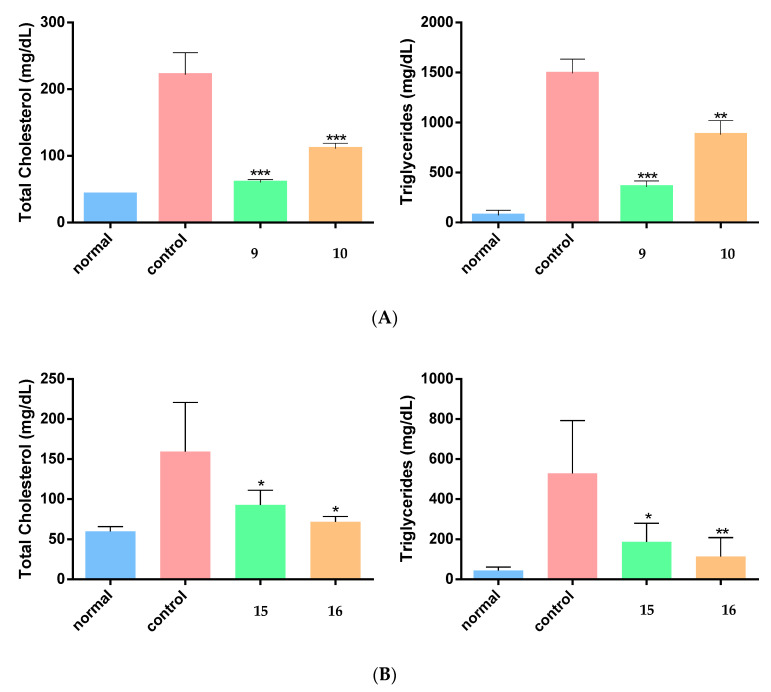
Effect of (**A**) compounds **9** and **10**; and (**B**) compounds **15** and **16** on total cholesterol and triglyceride levels of experimentally induced hyperlipidemic animals. Significant difference from (tyloxapol-treated) control: * *p* < 0.02, ** *p* < 0.002, *** *p* < 0.0001.

Finally, taking into account the hypolipidemic effect shown for several known NSAIDs [27], we evaluated the selected derivatives **9**, **10**, **15** and **16** in an experimentally induced hyperlipidemic mouse model. These derivatives were selected for their combined anti-inflammatory activity, in vitro and in vivo, by being potent LOX-3 inhibitors as well as demonstrating in vivo paw edema inhibition. Acute hyperlipidemia was achieved, *i.p.*, by the administration of tyloxapol (Triton WR 1339) [27]. The antihyperlipidemic effect of the tested compounds was evaluated as the decrease in total cholesterol (TC) and triglyceride (TG) levels in plasma 24 h after tyloxapol administration [26]. The results are shown in Figure 4A,B. Compounds **9** and **10** reduced TC levels in plasma by 73% and 50%, respectively, and TG levels by 76% and 41%, respectively. Compounds **15** and **16** also demonstrated good hypolipidemic activity by reducing TC levels by 42% and 55%, respectively, and TG levels by 65% and 78%, respectively.

It is worth mentioning that preliminary experiments indicated that these ester derivatives are quite stable and do not readily hydrolyze in the presence of aqueous conditions or in plasma (results not shown). Our estimation, in accordance with the literature related to ester-drug hydrolysis (e.g., aspirin or remdesivir), is that these derivatives act and deliver their activity as a whole; they do not seem to function as a prodrug of the parent NSAID which, in most cases, is less active than the new derivative.

### 2.4. In Silico Studies

#### Molecular Docking Simulations

In order to assess the theoretical affinity to LOX-3 for all new synthesized compounds, molecular docking simulations were run and compared with known crystallographic structures of LOX-3 in complex with 13(*S*)-hydroperoxy-9(*Z*),11(*E*)-octadecadienoic acid (13S-Hpode) (PDB id 1IK3) [28] and epigallocathechin (EPG) (PDB id 1JNQ) [29]. Induced fit docking was run for each derivative (see Materials and Methods for details). This procedure was intended to fine tune the aminoacid side chains and backbone movements upon ligand binding. All compounds were docked, and their binding scores were measured based on GlideScore as implemented on Schrodinger Suite 2018. For compounds with an IC_50_ lower than 100 μΜ, the predicted Glide Scores were between −10 and −13 Kcal/mol, while for the rest of the compounds, including almost all parent NSAIDs (a-n), the predicted glide scores were between −7.5 and −12 Kcal/mol. Since the glide score is based on an empirical scoring function and may introduce moderate or even large errors on binding affinity, we simply focused on visual inspection and comparison with the known binders 13S-Hpode and EPG for some of the potent synthesized compounds, i.e., **15**, **10**, **16** and **1**.

**Figure 5:**
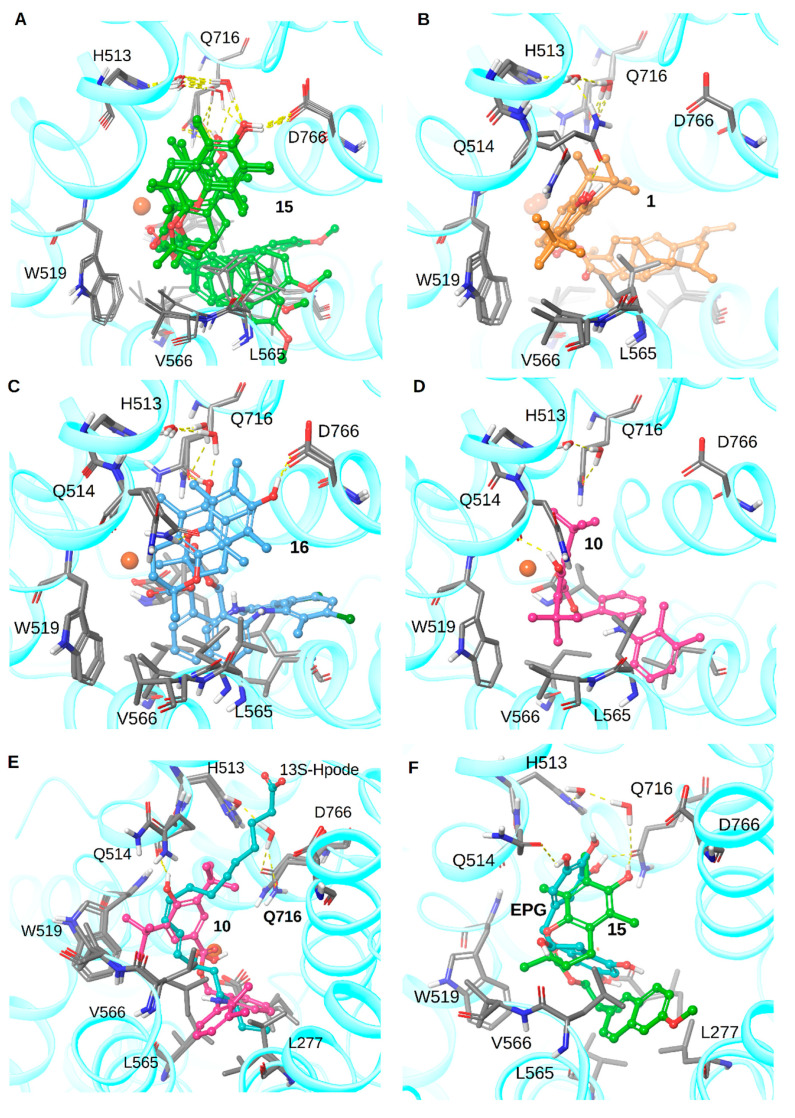
Docking poses of compounds **15** (**A**), **1** (**B**), **16** (**C**), and **10** (**D**) in the binding pocket of LOX-3. Superimposed docking pose of compound **10** and crystallographic binding mode of 13S-Hpode (original PDB entry) (**E**) and superimposed docking pose of compound **15** and crystallographic binding mode of EPG (original PDB entry) (**F**) as seen in the same binding pocket. Hydrogen bonds are depicted with yellow dashed lines.

The binding pocket of LOX-3 appears as an L-shaped space which is large enough to accommodate 13S-Hpode. Molecules able to adopt an approximate “L” shape with a 90-degree angle within their molecular skeleton could be appropriately accommodated in a similar fashion to 13S-Hpode or EPG (see also Supplementary Figures S2 and S3). The carboxylic acid of 13S-Hpode lies among hydrophilic residues R726 and D766 which are connected via a hydrogen bond network with two crystallographic water molecules, while the iron site lies in the middle of the substrate and the hydrophobic tail is found inside a hydrophobic pocket among residues L273, L277, I557, L565, L566, I772, and L773 (Figure 5).

All four diastereoisomers of compound **15** appeared to dock in a similar fashion where the phenolic OH group was oriented to Q514 and D766. It must be noted that the two crystallographic water molecules mentioned earlier were shifted next to Q514 and D766 stabilizing the binding mode of **15**. Moreover, the ester group was placed near the iron site. The hydrophobic sidechain was oriented to the hydrophobic pocket (Figure 5A). 

Compound **1** also was shown to dock in a similar way, where the OH group was placed near Q514. The two water molecules were further shifted, forming a hydrogen bond network with H513and Q716 (Figure 5B). Furthermore, the hydrophobic sidechain was oriented towards the hydrophobic pocket while the ester group was placed next to iron site (Figure 5B). 

Compound **16** showed two binding modes where the OH group formed a hydrogen bond with D766 or Q514, while the rest of the molecule was buried in the hydrophobic pocket with the ester group found again next to the iron site (Figure 5C). 

Finally, compound **10** was docked at a lower position compared to **15**, **1** and **16** probably due to its smaller volume. However, the OH group still formed a hydrogen bond with Q514, the ester group was found next to the iron site, and the hydrophobic tail was found buried inside the hydrophobic pocket (Figure 5D). A general observation from the docking simulations that can be made is that the ester group and the hydrophobic sidechain seem to play a major role in binding while the OH group is free to interact with hydrophilic residues while the water molecules are following the ligand in order to stabilize the binding and enhance affinity. When comparing the binding mode with the known co-crystalized structures, we can observe that compound **10** is superimposed very close to the crystallographically observed “natural” complex with 13S-Hpode (Figure 5E) and compound **15** is very close to the crystallographically observed “natural” complex with EPG (Figure 5F).

## 3. Materials and Methods 

### 3.1. Chemical Synthesis

#### 3.1.1. General Synthesis of Compounds **b**, **d**, **f**, **h**, **j** and **l**

To a cooled (0 °C) solution of 1 mmol of **a**, **c**, **e**, **g**, **i**, or **k** in dry dichloromethane, 1.1 mmol *N,N*-dicyclohexylcarbodiimide was added and the reaction mixture was stirred at 0 °C for 20–90 min. The mixture was then brought to room temperature and 10 mmol absolute ethanol was added dropwise. After the addition, the reaction mixture was heated at reflux for 4–24 h, after which it was diluted with dichloromethane, washed with water, NaHCO_3_ 5% solution and brine. The organic phase was dried over anhydrous sodium sulfate and evaporated to dryness. The crude product was purified by flash chromatography to give an ethyl ester of **a**, **c**, **e**, **g**, **i**, and **k**, respectively. Subsequently, 1 mmol of the respective ethyl ester was dissolved in dry diethylether and was added dropwise to a suspension of 1.2 mmol lithium aluminum hydride in dry diethylether, under Argon. The reaction mixture was stirred at room temperature for 1–2 h and then diluted with ethyl acetate and filtered. The precipitate was thoroughly washed with diethylether. The filtrate was diluted with diethylether and washed with H_2_SO_4_ 15% solution, water, NaHCO_3_ 5% solution and water, dried over anhydrous sodium sulfate and evaporated to dryness. The crude product was purified by flash chromatography to give pure **b**, **d**, **f**, **h**, **j**, and **l**.

*2-(4-Isobutylphenyl)propanoic acid* (**b**) Total yield: 62%, colorless oil. ^1^H-NMR (CDCl_3_, 200 MHz), δ (ppm): 0.90 (d, *J* = 6.8 Hz, 6Hz, -CH(CH_3_)_2_), 1.26 (d, *J* = 7.2 Hz, 3H, -CH(CH_3_)), 1.36 (brs, 1H, -OH), 1.85 (sep, *J* = 6.4 Hz, 1H, -CH(CH_3_)_2_), 2.45 (d, *J* = 7.2 Hz, 2H, -CH-CH_2_-), 2.93 (sex, *J* = 6.8 Hz, 1H, -CH(CH_3_)), 3.68 (d, *J* = 4.4 Hz, 2H, -CH_2_OH), 7.11–7.15 (4H, aromatic).

*2-(6-methoxynaphthalen-2-yl)propan-1-ol* (**d**) Total yield: 84%, off-white solid. m.p.: 81.9–82.9 °C. ^1^H NMR (400 MHz, CDCl_3_), δ (ppm): 1.35 (d, 3H, -CH(CH_3_)), 1.48 (brs, 1H, -OH), 3.09 (sex, *J* = 13.8, 6.9 Hz, 1H, -CH(CH_3_)), 3.78 (d, *J* = 6.8 Hz, 2H, -CH_2_OH), 3.92 (s, 3H, -OCH_3_), 7.14–7.71 (6H, aromatic). 

*(2-((3-chloro-2-methylphenyl)amino)phenyl)methanol* (**f**) Total yield: 95%, orange solid. m.p.: 48.4–48.7 °C. ^1^H NMR (400 MHz, CDCl_3_) δ (ppm): 2.34 (s, 3H, -CH_3_), 4.77 (s, 2H, -CH_2_OH), 6.87–7.24 (7H, aromatic). 

*(2-((3-(trifluoromethyl)phenyl)amino)phenyl)methanol* (**h**) Total yield: 48%, yellow oil. ^1^H NMR (400 MHz, CDCl_3_), δ (ppm): 1.77 (brs, 1H, -OH), 4.73 (s, 2H, -CH_2_OH), 6.97–7.36 (8H, aromatic). 

*(2-((2,3-dimethylphenyl)amino)phenyl)methanol* (**j**) Total yield: 56%, yellow solid. m.p.: 58.2–61 °C. ^1^H-NMR (400 MHz, CDCl_3_), δ (ppm): 1.29 (s, 1H, -OH), 2.14 (s, 3H, 2-CH_3_), 2.33 (s, 3H, 3-CH_3_), 4.70 (s, 2H, -CH_2_-), 6.79–7.05 (7H, aromatic).

*2-(2-((2,6-dimethylphenyl)amino)phenyl)ethan-1-ol* (**l**) Total yield: 19%, orange solid. m.p.: 105.7–106.2 °C. ^1^H NMR (400 MHz, CDCl_3_), δ (ppm): 3.03 (t, *J* = 5.8 Hz, 2H, -CH_2_CH_2_OH), 4.04 (t, *J* = 5.8 Hz, 2H, CH_2_CH_2_OH), 6.48–7.34 (7H, aromatic). 

#### 3.1.2. Synthesis of (4-chlorophenyl)(3-(2-hydroxyethyl)-5-methoxy-2-methyl-1H-indol-1-yl)methanone (**n**)

To a cooled (−20 °C) solution of 0.100 g (0.28 mmol) of indomethacin (**m**) in 2 mL dry THF, 0.310 mL (0.31 mmol) of borane in THF 1M was added dropwise. The reaction mixture was heated at 0 °C and stirred for 5 days. Then, it was diluted with acetic acid and evaporated to dryness. The residues were diluted in ethyl acetate and washed with NaHCO_3_ 5% solution and brine. The organic phase was dried over anhydrous sodium sulfate and evaporated to dryness to give a pure product (0.095 g, 99% yield, yellow solid). m.p.: 86.7–87.2 °C. ^1^H NMR (400 MHz, CDCl_3_), δ (ppm): 2.37 (s, 3H, -CH_3_), 2.94 (t, *J* = 6.6 Hz, 2H, -CH_2_CH_2_OH), 3.83 (s, 3H, -OCH_3_), 3.86 (t, *J* = 6.7 Hz, 2H, -CH_2_OH), 6.66–7.65 (7H, aromatic). 

#### 3.1.3. General Synthesis of Compounds **1**–**13**

To a cooled (0 °C) solution of 1 mmol of the respective carboxylic acid and 1.3 mmol of the corresponding alcohol in dry dichloromethane, 1.1 mmol of *N,N*-dicyclohexylcarbodiimide and a catalytic amount of dimethylaminopyridine were added. The reaction mixture was stirred at room temperature for 24 h and then filtered. The filtrate was diluted with dichloromethane and washed with water. The organic phase was dried over anhydrous sodium sulfate and evaporated to dryness. The crude product was purified by flash chromatography, to give the final compounds **1**–**13**.

*3,5-di-tert-butyl-4-hydroxybenzyl 2-(4-isobutylphenyl)propanoate* (**1**) Yield: 60%, yellow semisolid. ^1^H-NMR (400 MHz, CDCl_3_ ), δ (ppm): 0.90 (d, *J* = 6.8 Hz, 6Hz, -CH(CH_3_)_2_), 1.41 (s, 18H, 3,5-C(CH_3_)_3_), 1.51 (d, 3H, -CH(CH_3_)), 1.84 (m, 1H, -CH(CH_3_)_2_), 2.45 (d, *J* = 7.2 Hz, 2H, -CH_2_CH(CH_3_)_2_), 3.74 (m, 1H, -CH(CH_3_)), 5.02 (dd, 2H, -OCH_2_-), 5.22 (s, 1H, -OH), 7.05–7.26 (6H, aromatic). ^13^C NMR (101 MHz, CDCl_3_), δ (ppm): 18.65 (-CH(CH_3_)), 22.42 (-CH(CH_3_)_2_), 30.20 (3,5-C(CH_3_)_3_, -CH(CH_3_)_2_), 34.28 (3,5-C(CH_3_)_3_), 45.06 (-CH_2_CH(CH_3_)_2_), 45.17 (-CH(CH_3_)), 67.16 (-OCH_2_-), 125.19 (2,6-hydroxybezyl), 126.68 (1-hydroxybenzyl), 127.22 (3,5-phenyl), 129.31 (2,6-phenyl), 135.90 (3,5-hydroxybenzyl), 137.82 (1-phenyl), 140.46 (4-phenyl), 153.76 (4-hydroxybenzyl), 174.75 (C=O). 

*2-(4-isobutylphenyl)propyl 3,5-di-tert-butyl-4-hydroxybenzoate* (**2**) Yield: 50%, yellow semisolid. ^1^H-NMR (400 MHz, CDCl_3_), δ (ppm): 0.90 (d, *J* = 6.8 Hz, 6Hz, -CH(CH_3_)_2_), 1.36–1.56 (m, 21H, 3,5-C(CH_3_)_3_), -CH(CH_3_)), 1.85 (m, 1H, -CH(CH_3_)_2_), 2.45 (d, *J* = 7.2 Hz, 2H, -CH_2_CH(CH_3_)_2_), 3.20 (m, 1H, -CH(CH_3_)), 4.34 (d, *J* = 4.4 Hz, 2H, -OCH_2_-), 5.65 (s, 1H, -OH), 7.08–7.85 (6H, aromatic).

*3,5-di-tert-butyl-4-hydroxybenzyl 2-(6-methoxynaphthalen-2-yl)propanoate* (**3**) Yield: 81%, white solid. m.p.: 85–86 °C. ^1^H NMR (400 MHz, CDCl_3_), δ (ppm): 1.40 (s, 18H, 3,5-C(CH_3_)_3_), 1.62 (d, *J* = 7.1 Hz, 3H, -CH(CH_3_)), 3.95 (s, 3H, -OCH_3_), 3.89–4.07 (m, 1H, -CH(CH_3_)), 5.06 (s, 2H, -OCH_2_-), 5.26 (brs, 1H, -OH), 7.08–7.65 (8H, aromatic). ^13^C NMR (101 MHz, CDCl_3_), δ (ppm): 20.15 (-CH(CH_3_)), 30.85 (3,5-C(CH_3_)_3_), 32.45 (3,5-C(CH_3_)_3_), 45.39 (-CH(CH_3_)), 56.92 (-OCH_3_), 69.44 (-CH_2_O-), 107.87 (5-naphthalen), 116.23 (7-naphthalen), 123.34 (1-phenyl), 124.65 (2,6-phenyl), 128.65 (1-naphthalen), 129.42 (4-naphthalen), 130.73 (3-naphthalen), 132.91 (8-naphthalen), 133.41 (8a-naphthalen), 135.67 (4a-naphthalen), 133.87 (3,5-phenyl), 139.25 (2-naphthalen), 151.03 (4-phenyl), 158.48 (6-naphthalen), 176.93 (C=O).

*2-(6-methoxynaphthalen-2-yl)propyl 3,5-di-tert-butyl-4-hydroxybenzoate* (**4**) Yield: 39%, off-yellow solid. m.p.: 154.1–155 °C. ^1^H NMR (400 MHz, CDCl_3_), δ (ppm): 1.41 (s, 18H, 3,5-C(CH_3_)_3_), 1.47 (d, *J* = 7.0 Hz, 3H, -CH_3_), 3.38 (sex, *J* = 13.9, 6.9 Hz, 1H, -CH(CH_3_)), 3.92 (s, 3H, -OCH_3_), 4.43 (d, *J* = 6.9 Hz, 2H, -CH_2_O-), 5.62 (s, 1H, -OH), 7.11–7.82 (8H, aromatic). ^13^C NMR (101 MHz, CDCl_3_), δ (ppm): 17.95 (-CH(CH_3_)), 30.10 (3,5-C(CH_3_)_3_), 34.28 (3,5-C(CH_3_)_3_), 39.11 (-CH(CH_3_)), 55.32 (-OCH_3_), 69.55 (-CH_2_O-), 105.59 (5-naphthalen), 118.77 (7-naphthalen), 121.26 (1-benzoic), 125.65 (2,6-benzoic), 126.43 (1-naphthalen), 126.94 (4-naphthalen), 127.04 (3-naphthalen), 129.04 (8-naphthalen), 129.15 (8a-naphthalen), 133.51 (4a-naphthalen), 135.63 (3,5-benzoic), 138.70 (2-naphthalen), 157.39 (6-naphthalen), 158.11 (4-benzoic), 167.03 (C=O). 

*3,5-di-tert-butyl-4-hydroxybenzyl 2-((3-chloro-2-methylphenyl)amino)benzoate* (**5**) Yield: 10%, yellow semisolid. ^1^H NMR (400 MHz, CDCl_3_), δ (ppm): 1.46 (s, 18H, 3,5-C(CH_3_)_3_), 2.32 (s, 3H, -CH_3_), 5.26 (s, 2H, -OCH_2_-), 5.29 (s, 1H, -NH-), 6.71–8.02 (9H, aromatic), 9.34 (brs, 1H, -OH). ^13^C NMR (101 MHz, CDCl_3_), δ (ppm): 15.06 (2-CH_3_), 30.30 (3,5-C(CH_3_)_3_), 34.30 (3,5-C(CH_3_)_3_), 72.68 (-OCH_2_-), 110.07 (3-benzoic), 113.86 (5-benzoic), 117.03 (4-phenyl), 123.47 (2,6-hydroxybenzyl), 125.11 (1-benzoic), 126.11 (6-phenyl), 126.94 (5-phenyl), 129.08 (1-hydroxybenzyl), 131.99 (6-benzoic), 132.07 (2-phenyl), 135.12 (4-benzoic), 135.67 (3-phenyl), 135.80 (3,5-hydroxybenzyl), 140.07 (1-phenyl), 149.28 (2-benzoic), 153.39 (4-hydroxybenzyl), 167.20 (C=O)

*2-((3-chloro-2-methylphenyl)amino)benzyl 3,5-di-tert-butyl-4-hydroxybenzoate* (**6**) Yield: 67%, white solid. m.p.: 141.1–141.8 °C. ^1^H NMR (400 MHz, CDCl_3_), δ (ppm): 1.46 (s, 18H, 3,5-C(CH_3_)_3_), 2.33 (s, 3H, -CH_3_), 5.39 (s, 2H, -OCH_2_-), 5.71 (s, 1H, -OH), 6.62 (s, 1H, -NH-), 6.95–7.00 (m, *J* = 7.4, 1.1 Hz, 1H, aromatic), 7.03 (d, *J* = 6.6 Hz, 4H, aromatic), 7.22–7.25 (m, 1H, aromatic), 7.47 (dd, *J* = 7.6, 1.5 Hz, 1H, aromatic), 7.92 (s, 2H, aromatic). ^13^C NMR (101 MHz, CDCl_3_), δ (ppm): 14.60 (2-CH_3_), 30.15 (3,5-C(CH_3_)_3_), 34.35 (3,5-C(CH_3_)_3_), 64.53 (-OCH_2_-), 117.19 (4-phenyl), 118.71 (1-benzyl), 120.77 (6-benzyl), 121.25 (1-benzoic), 122.52 (3-benzyl), 125.30 (2,6-benzoic), 126.39 (6-phenyl), 126.88 (4,5-benzyl), 129.72 (5-phenyl), 131.98 (2-phenyl), 135.36 (3-phenyl), 135.86 (3,5-benzoic), 143.17 (2-benzyl), 143.21 (1-phenyl), 158.49 (4-benzoic), 167.34 (C=O). 

*3,5-di-tert-butyl-4-hydroxybenzyl 2-((3-(trifluoromethyl)phenyl)amino)benzoate* (**7**) Yield: 38%, orange solid. m.p.: 87.2–88.0 °C. ^1^H NMR (400 MHz, CDCl_3_), δ (ppm): 1.36 (s, 9H, 3,5-C(CH_3_)_3_), 1.42 (s, 9H, 3,5-C(CH_3_)_3_), 4.64 (s, 1H, 1/2xCH_2_O-), 5.01 (s, 1H, 1/2xCH_2_O-), 6.64 (dd, *J* = 8.4, 2.4 Hz, 1H, aromatic), 6.83 (s, 1H, aromatic), 6.90 (d, *J* = 7.6 Hz, 1H, aromatic), 7.03 (s, 1H, aromatic), 7.11–7.16 (m, 2H, aromatic), 7.29–7.34 (m, 1H, aromatic), 7.49 (td, *J* = 7.7, 1.7 Hz, 1H, aromatic), 7.73 (s, 1H, aromatic), 7.95 (dd, *J* = 7.8, 1.6 Hz, 1H, aromatic). ^13^C NMR (101 MHz, CDCl_3_), δ (ppm): 30.16 (3,5-C(CH_3_)_3_), 34.27 (3,5-C(CH_3_)_3_), 56.97 (-OCH_2_-), 117.19 (5-benzoic), 124.13 (2,6-hydroxybenzyl), 125.65 (1-benzoic), 126.20 (-CF_3_), 126.46 (2-phenyl), 127.68 (1-hydroxybenzyl), 128.20 (4-phenyl), 128.63 (6-phenyl), 129.06 (3-benzoic), 129.98 (5-phenyl), 131.03 (6-benzoic), 132.27 (3-phenyl), 133.22 (4-benzoic), 135.94 (3,5-hydroxybenzyl), 146.24 (1-phenyl), 149.02 (2-benzoic), 154.09 (4-hydroxybenzyl), 191.84 (C=O).

*2-((3-(trifluoromethyl)phenyl)amino)benzyl 3,5-di-tert-butyl-4-hydroxybenzoate* (**8**) Yield: 60%, white solid. m.p.: 119.9–120.5 °C. ^1^H NMR (400 MHz, CDCl_3_), δ (ppm): 1.46 (s, 18H, 3,5-C(CH_3_)_3_), 5.37 (s, 2H, -OCH_2_-), 5.71 (s, 1H, -NH-), 7.04–7.91 (10H, aromatic). ^13^C NMR (101 MHz, CDCl_3_), δ (ppm): 30.12 (3,5-C(CH_3_)_3_), 34.35 (3,5-C(CH_3_)_3_), 64.06 (-OCH_2_-), 113.23 (4-phenyl), 113.27 (6-phenyl), 116.57 (2-phenyl), 119.38 (-CF_3_), 119.80 (3-benzyl), 120.64 (1-benzoic), 122.45 (2-benzyl), 126.64 (6-benzyl), 127.24 (2,6-benzoic), 129.79 (4,5-benzyl), 129.85 (5-phenyl), 132.24 (3-phenyl), 135.91 (3,5-benzoic), 141.43 (1-phenyl), 144.37 (1-benzyl), 158.57 (4-benzoic), 167.50 (C=O).

*3,5-di-tert-butyl-4-hydroxybenzyl 2-((2,3-dimethylphenyl)amino)benzoate* (**9**) Yield: 10%, yellow semisolid. ^1^H-NMR (400 MHz, CDCl_3_), δ (ppm): 1.40 (s, 18H, 3,5-C(CH_3_)_3_), 2.11 (s, 3H, 2-CH_3_), 2.27 (s, 3H, 3-CH_3_), 5.20 (s, 2H, -OCH_2_-), 6.45–7.95 (10 H, aromatic and –OH), 9.21 (s, 1H, -NH-). ^13^C-NMR (200 MHz, CDCl_3_), δ (ppm): 13.93 (2-CH_3_), 20.59 (3-CH_3_), 30.24 (3,5-C(CH_3_)_3_), 34.32 (3,5-C(CH_3_)_3_), 67.13 (-OCH_2_-), 113.65–134.06 (aromatic). 

*2-((2,3-dimethylphenyl)amino)benzyl 3,5-di-tert-butyl-4-hydroxybenzoate* (**10**) Yield: 68%, white solid. m.p.: 136.3–136.9 °C. ^1^H-NMR (400 MHz, CDCl_3_), δ (ppm): 1.46 (s, 18H, 3,5-C(CH_3_)_3_), 2.15 (s, 3H, 2-CH_3_), 2.33 (s, 3H, 3-CH_3_), 5.41 (s, 2H, -CH_2_-), 5.70 (s, 1H, -OH), 6.39 (s, 1H, -NH-), 6.90–7.39 (9H, aromatic). ^13^C-NMR (200 MHz, CDCl_3_), δ (ppm): 13.75 (2-CH_3_), 20.70 (3-CH_3_), 30.16 (3,5-C(CH_3_)_3_), 34.35 (3,5-C(CH_3_)_3_), 64.90 (-OCH_2_-), 117.10–158.40 (aromatic), 167.27 (C=O). 

*3,5-di-tert-butyl-4-hydroxybenzyl 2-(2-((2,6-dichlorophenyl)amino)phenyl)acetate* (**11**) Yield: 65%, yellow solid. m.p.: 149.0–149.7 °C. ^1^H NMR (400 MHz, CDCl_3_), δ (ppm): 1.42 (s, 18H, 3,5-C(CH_3_)_3_), 3.85 (s, 2H, -CH_2_C=O), 5.09 (s, 2H, -CH_2_O-), 5.27 (s, 1H, -NH-), 6.55–7.33 (7H, aromatic). ^13^C NMR (101 MHz, CDCl_3_), δ (ppm): 30.17 (3,5-C(CH_3_)_3_), 34.33 (3,5-C(CH_3_)_3_), 38.83 (-CH_2_C=O), 68.06 (-OCH_2_-), 118.34 (5-phenylacetate), 122.04 (4-phenyl), 123.97 (1-phenylacetate), 124.50 (2-phenylacetate), 125.96 (2,6-hydroxybenzyl), 126.16 (1-hydroxybenzyl), 127.94 (4-phenylacetate), 128.87 (3,6-phenylacetate), 129.52 (3,5-phenyl), 130.94 (1-phenyl), 136.07 (3,5-hydroxybenzyl), 137.91 (6-phenyl), 142.82 (2-phenyl), 154.13 (4-hydroxybenzyl), 172.37 (C=O).

*2-((2,6-dichlorophenyl)amino)phenethyl 3,5-di-tert-butyl-4-hydroxybenzoate* (**12**) Yield: 49%, white solid. m.p.: 147.8–149.2 °C. ^1^H NMR (400 MHz, CDCl_3_), δ (ppm): 1.45 (s, 18H, 3,5-C(CH_3_)_3_), 3.20 (t, *J* = 7.5 Hz, 2H, -CH_2_CH_2_O-), 4.60 (t, *J* = 7.5 Hz, 2H, -CH_2_CH_2_O-), 5.66 (s, 1H, -NH-), 6.08 (brs, 1H, -OH), 6.45–7.89 (9H, aromatic). ^13^C NMR (101 MHz, CDCl_3_), δ (ppm): 30.15 (3,5-C(CH_3_)_3_), 31.48 (-OCH_2_CH_2_-), 34.35 (3,5-C(CH_3_)_3_), 63.37 (-OCH_2_CH_2_-), 116.31 (5-phenethyl), 121.21 (4-phenyl), 121.29 (1-benzoic), 124.96 (1-phenethyl), 125.61 (2-phenethyl), 127.17 (2,6-benzoic), 127.39 (4-phenethyl), 128.86 (3,6-phenethyl), 130.73 (1-phenyl), 131.11 (3,5-phenyl), 135.69 (3,5-benzoic), 137.36 (6-phenyl), 142.10 (2-phenyl), 158.24 (4-benzoic), 167.40 (C=O). 

*3,5-di-tert-butyl-4-hydroxybenzyl 2-(1-(4-chlorobenzoyl)-5-methoxy-2-methyl-1H-indol-3-yl)acetate* (**13**) Yield: 74%, yellow solid. m.p.: 149.3–149.9 °C. ^1^H NMR (400 MHz, CDCl_3_), δ (ppm): 1.40 (s, 18H, 3,5-C(CH_3_)_3_), 2.38 (s, 3H, -CH_3_), 3.70 (s, 2H, -CH_2_C=O), 3.71 (s, 3H, -OCH_3_), 5.05 (s, 2H, -CH_2_O-), 5.27 (s, 1H, -OH), 6.64–7.67 (9H, aromatic). ^13^C NMR (101 MHz, CDCl_3_), δ (ppm): 13.43 (2-CH_3_), 30.18 (3,5-C(CH_3_)_3_), 30.56 (-CH_2_C=O), 34.28 (3,5-C(CH_3_)_3_), 55.51 (-OCH_3_), 67.69 (-OCH_2_-), 101.32 (4-indole), 111.76 (6-indole), 112.72 (7-indole), 114.92 (3-indole), 125.70 (2,6-hydroxybenzyl), 126.32 (1-hydroxybenzyl), 129.11 (3,5-phenyl), 130.66 (7a-indole), 130.79 (1-phenyl), 131.19 (2,6-phenyl), 133.94 (2-indole), 135.89 (3a-indole), 136.03 (3,5-hydroxybenzyl), 139.23 (4-phenyl), 154.01 (5-indole), 155.65 (4-hydroxybenzyl), 168.28 (-N-C=O)), 170.84 (-CH_2_-C=O-). 

#### 3.1.4. General Synthesis of Compounds **14**–**17**

To a cooled (0 °C) solution of 1 mmol of 6-hydroxy-2,5,7,8-tetramethylchroman-2-carboxylic acid in dry dichloromethane, 3 mmol of the corresponding alcohol b, d, f or n, 1.1 mmol *N,N*-dicyclohexylcarbodiimide and a catalytic amount of dimethylaminopyridine were added. The reaction mixture was stirred at 0 °C for 5 min and at room temperature for 5 h (or 3 days for compound **17**) and then filtered. The filtrate was diluted with dichloromethane, washed with 0.5N HCl solution and NaHCO_3_ 5% solution, dried over anhydrous sodium sulfate and evaporated to dryness. The crude product was purified by flash chromatography to yield the final compounds **14**–**17**.

*2-(4-isobutylphenyl)propyl6-hydroxy-2,5,7,8-tetramethylchromane-2-carboxylate* (**14**) Yield: 94%, white solid. m.p.: 69.2–70.0 °C. ^1^H NMR (400 MHz, CDCl_3_), δ (ppm): 0.94 (d, 6H, -CH(CH_3_)_2_)), 1.21 (d, 3H, -CH(CH_3_)), 1.33 (dd, *J* = 9.0, 5.7 Hz, 1H, -CH(CH_3_)_2_), 1.56 (d, *J* = 3.8 Hz, 3H, 2-CH_3_), 1.78–1.93 (m, *J* = 19.3, 8.6, 4.5 Hz, 2H, 3-CH_2_), 2.06 (d, *J* = 5.4 Hz, 3H, 8-CH_3_), 2.20 (d, *J* = 7.8 Hz, 6H, 5,7-CH_3_), 2.30–2.42 (m, 1H, 1/2x 4-CH_2_), 2.47 (d, *J* = 10.7 Hz, 2H, -CH_2_CH(CH_3_)_2_), 2.52–2.64 (m, 1H, 1/2 × 4-CH_2_), 3.04 (sex, 1H, -CH(CH_3_)), 4.07–4.16 (m, *J* = 8.8, 6.5, 2.5 Hz, 1H, 1/2 × -OCH_2_-), 4.19–4.30 (m, 1H, 1/2 × -OCH_2_-), 4.36 (brs, 1H, -OH), 7.09 (d, *J* = 2.4 Hz, 4H, aromatic). ^13^C NMR (101 MHz, CDCl_3_), δ (ppm): 11.22 (5-CH_3_), 11.82 (7-CH_3_), 12.21 (8-CH_3_), 17.74 (-CH(CH_3_)), 20.84 (-C(CH_3_)), 22.38 (-CH(CH_3_)_2_), 25.50 (3-CH_2_), 30.23 ((-CH(CH_3_)_2_), 30.60 (4-CH_2_), 38.55 (-CH(CH_3_)), 45.04 (-CH_2_CH(CH_3_)_2_), 70.04 (-OCH_2_-), 77.00 (-C(CH_3_)), 116.89 (4a-chromane), 118.35 (5-chromane), 121.13 (7-chromane), 122.49 (8-chromane), 126.96 (3,5-phenyl), 129.14 (2,6-phenyl), 139.99 (4-phenyl), 140.26 (1-phenyl), 145.24 (6-chromane), 145.64 (8a-chromane), 173.83 (C=O). 

*2-(6-methoxynaphthalen-2-yl)propyl 6-hydroxy-2,5,7,8-tetramethylchromane-2-carboxylate* (**15**) Yield: 94%, yellow semisolid. ^1^H NMR (400 MHz, CDCl_3_), δ (ppm): 1.26 (d, *J* = 7.0 Hz, 3H, -CH(CH_3_)), 1.52 (s, 3H, 2-CH_3_), 1.88 (s, 1H, 1/2 × 3-CH_2_), 1.95 (s, 1H, 1/2 × 3-CH_2_), 2.12 (dd, *J* = 13.0, 8.8 Hz, 9H, 5,7,8-CH_3_), 2.26–2.40 (m, 2H, 4-CH_2_), 3.18 (dq, *J* = 13.8, 7.0 Hz, 1H, -CH(CH_3_)), 3.92 (s, 3H, -OCH_3_), 4.12–4.20 (m, 1H, 1/2 × -OCH_2_-), 4.32 (dt, *J* = 10.8, 6.8 Hz, 1H, 1/2 × -OCH_2_), 7.13–7.65 (6H, aromatic). ^13^C NMR (101 MHz, CDCl_3_), δ (ppm): 11.77 (5-CH_3_), 12.18 (7-CH_3_), 14.21 (8-CH_3_), 17.86 (-CH(CH_3_)), 21.06 (-C(CH_3_)), 25.53 (3-CH_2_), 30.60 (4-CH_2_), 38.88 (-CH(CH_3_)), 55.29 (-OCH_3_), 69.75 (-CH_2_O-), 77.22 (-C(CH_3_)), 105.58 (5-naphthalen), 116.8 (4a-chromane), 118.36 (5-chromane), 118.84 (7-naphthalen), 121.18 (7-chromane), 125.38 (8-chromane), 125.56 (1-naphthalen), 126.23 (4-naphthalen), 126.94 (3-naphthalen), 128.96 (8-naphthalen), 129.11 (8a-naphthalen), 133.50 (4a-naphthalen), 138.20 (2-naphthalen), 145.15 (6-chromane), 145.58 (8a-chromane), 157.44 (6-naphthalen), 171.18 (C=O). 

*2-((3-chloro-2-methylphenyl)amino)benzyl 6-hydroxy-2,5,7,8-tetramethylchromane-2-carboxylate* (**16**) Yield: 56%, off-yellow solid. m.p.: 150.4–151.3 °C. ^1^H NMR (400 MHz, CDCl_3_), δ (ppm): 1.59 (s, 3H, 2-CH_3_ on chromane), 1.80–1.90 (m, *J* = 12.0, 10.7, 6.3 Hz, 1H, 1/2 × 3-CH_2_), 1.95 (s, 3H, 2-CH_3_ on phenyl), 2.15 (s, 6H, 5,7-CH_3_), 2.21 (s, 3H, 8-CH_3_), 2.28–2.43 (m, 2H, 4-CH_2_), 2.51–2.58 (m, 1H, 1/2 × 3-CH_2_), 4.20 (s, 1H, -NH-), 5.14 (s, 2H, -OCH_2_), 6.11 (brs, 1H, -OH), 6.78–7.19 (7H, aromatic). ^13^C NMR (101 MHz, CDCl_3_), δ (ppm): 11.16 (5-CH_3_), 11.84 (7-CH_3_), 12.20 (8-CH_3_), 14.42 (2-CH_3_ on phenyl), 20.80 (2-CH_3_ on chromane), 25.28 (3-CH_2_), 30.85 (4-CH_2_), 64.50 (-OCH_2_), 77.14 (-C(CH_3_)), 116.88 (4a-chromane), 117.36 (4-phenyl), 118.36 (5-chromane), 119.20 (1-benzyl), 121.20 (5-benzyl), 121.52 (7-chromane), 122.54 (6-benzyl), 122.68 (8-chromane), 125.07 (6-phenyl), 126.52 (3-benzyl), 126.90 (4-benzyl), 129.78 (5-phenyl), 131.53 (2-phenyl), 135.35 (3-phenyl), 142.80 (2-benzyl), 143.23 (1-phenyl), 145.36 (6-chromane), 145.56 (8a-chromane), 174.33 (C=O). 

*2-(1-(4-chlorobenzoyl)-5-methoxy-2-methyl-1H-indol-3-yl)ethyl 6-hydroxy-2,5,7,8-tetramethylchromane-2-carboxylate* (**17**) Yield: 76%, yellow semisolid. ^1^H NMR (400 MHz, CDCl_3_), δ (ppm): 1.56 (s, 3H, 2-CH_3_ on chromane), 1.76–1.89 (m, 1H, 1/2 × 3-CH_2_), 2.00 (s, 3H, 8-CH_3_), 2.15 (d, *J* = 10.5 Hz, 6H, 5,7-CH_3_), 2.31 (s, 3H, 2-CH_3_ on indole), 2.37 (dd, *J* = 14.5, 7.2 Hz, 2H, 4-CH_2_), 2.50–2.62 (m, 1H, 1/2 × 3-CH_2_), 2.89–3.01 (m, 2H, -OCH_2_CH_2_-), 3.84 (s, 3H, -OCH_3_), 4.21–4.34 (m, 2H, -OCH_2_-), 4.38 (brs, 1H, -OH), 6.67–7.63 (7H, aromatic). ^13^C NMR (101 MHz, CDCl_3_), δ (ppm): 11.22 (5-CH_3_), 11.83 (7-CH_3_), 12.22 (8-CH_3_), 13.28 (2-CH_3_ on indole), 20.83 (2-CH_3_ on chromane), 23.57 (-CH_2_CH_2_O-), 25.24 (3-CH_2_), 30.62 (4-CH_2_), 55.79 (-OCH_3_), 63.79 (-OCH_2_-), 76.96 (-C(CH_3_)), 101.30 (4-indole), 111.35 (6-indole), 114.99 (7-indole), 115.0 (3-indole), 116.85 (4a-chromane), 118.52 (5-chromane), 121.38 (7-chromane), 122.49 (8-chromane), 129.11 (3,5-phenyl), 130.79 (7a-indole), 130.95 (1-phenyl), 131.11 (2,6-phenyl), 133.99 (2-indole), 135.29 (3a-indole), 139.20 (4-phenyl), 145.29 (6-chromane), 145.52 (8a-chromane), 156.03 (5-indole), 168.29(-N-C=O), 173.85 (-C=O).

### 3.2. Pharmacological Evaluation

#### 3.2.1. LOX Inhibition

Lipoxygenase activity was determined using soybean lipoxygenase (250 U/mL) and sodium linoleate (100 μM) as substrate, in Tris–HCl buffer pH 9.0. The test compounds dissolved in methanol were added and the reaction was monitored for 5 min at 28 °C, recording absorbance at 234 nm. Each concentration was evaluated twice and the results were expressed as IC_50_ (μM) after incubation for 5 min [22].

#### 3.2.2. Edema Reduction

For the in vivo anti-inflammatory activity, Wistar male rats or C57BL/6 mice were injected with 0.025 mL carrageenan (2% w/v solution in saline) *i.d.* into the right hind paw, with the left paw serving as the control. The test compounds (300 μmol/kg) were given, *i.p.*, after the carrageenan injection, and 3.5 h later, the produced edema was estimated as the paw weight increase. Results are expressed as percentage of the reduction in paw edema and are the means for six animals (per compound) [22].

#### 3.2.3. Radical Scavenging of DPPH

Compounds dissolved in absolute methanol at concentrations of 25–400 μM were added to an equal volume of a methanolic solution of DPPH (final concentration 200 μM) at room temperature (22 ± 2 °C). Absorbance (517 nm) was recorded at different time intervals for 1 h and results were expressed as IC_50_ (μM) for DPPH after incubation for 30 min [3].

#### 3.2.4. Inhibition of Lipid Peroxidation

The incubation mixture contained heat-inactivated (90 °C for 90 s) liver microsomal fraction from untreated C57BL/6 mice (corresponding to 2.5 mg of hepatic protein per milliliter or 4 mM fatty acid residues), ascorbic acid (0.2 mM) in Tris–HCl/KCl buffer (50 mM/ 150 mM, pH 7.4), and the studied compounds dissolved in dimethyl sulfoxide (in different concentrations). The reaction was initiated by the addition of a freshly prepared FeSO_4_ solution (10 μM), and the mixture was incubated at 37 °C for 45 min. Aliquots were taken at various time intervals and lipid peroxidation was assessed by the spectrophotometric (535 against 600 nm) determination of the produced 2-thiobarbituric acid reactive material [22].

#### 3.2.5. Hypolipidemic Activity

An aqueous solution of Triton WR 1339 was given, *i.p.*, to mice (400 mg/kg of body weight) and one hour later the test compounds (84 μmol/kg of body weight), dissolved in saline or saline only were administered, *i.p.* After 24 h, blood was taken from the aorta/heart and used for the determination of plasma total cholesterol (TC) and triglyceride (TG) levels, using commercially available kits. Levels of plasma lipids were determined in duplicate while the values presented were the mean from eight animals (per compound) [22].

### 3.3. In Silico Studies

#### 3.3.1. Machine Learning Activity Classification

From the ChEMBL database, we selected all molecules with IC_50_ lower than 1000 nM against LOX-3 or DPPH. The resulting pool contained 586 (Class 1) and 469 compounds (Class 2), respectively. We created an additional decoy set (Class 3) with 500 compounds from each target with IC_50_ greater than 1000 nM. For all selected compounds, we calculated 277 molecular descriptors from Molecular Descriptors workflow as implemented on Schrodinger Suite 2018. MATLAB v2018b Classification Learner was utilized to select the most accurate machine learning algorithm for discrimination based on the prediction accuracy resulted. All mathematical calculations were run using the software package MATLAB v2018b developed by MathWorks, Portola Valley, CA, USA (http://www.mathworks.com, accessed on 21 July 2021).

#### 3.3.2. Molecular Docking Simulations

Molecular Docking Simulations were run using the crystal structure of LOX-3 in complex with 13(*S*)-hydroperoxy-9(*Z*),11(*E*)-octadecadienoic acid (13S-Hpode) (PDB id 1IK3). The complex was prepared based on the Protein Preparation Wizard as implemented on Maestro 11.5 (Schrödinger, LLC, New York, NY, 2018). All compounds were sketched using Maestro 11.5 Software and their 3D structure was prepared using the ligprep workflow as implemented on Schrodinger Suite 2018 (Schrödinger, LLC, New York, NY, 2018). For compounds having one or two chiral centers, all enantiomers or diastereoisomers (2 or 4, respectively) were generated and further utilized in our studies. Starting from the crystal structure in complex with 13(*S*)-hydroperoxy-9(*Z*),11(*E*)-octadecadienoic acid, (PDB id 1IK3) Induced Fit Docking was run for each derivative as implemented on Schrodinger Suite 2018. Initial Glide docking was run for each ligand using a softened potential and optional removal of side chains and the application of constraints. Secondly, prime side-chain prediction was run for each protein–ligand complex, on residues within a 5 Å radius. Finally, Glide redocking was run for each protein–ligand complex structure within 30 kcal/mol. The ligand was thus rigorously docked using default Glide settings, into the induced-fit protein structure. All low energy complexes were further compared by superposition with the initial crystallographic structure along with the crystallographic structure of LOX-3 in complex with epigallocatechin (EPG) (PDB id 1JNQ).

## 4. Conclusions

We designed, synthesized and evaluated new conjugates of commonly used NSAIDs (or their corresponding alcohols) with the antioxidant moieties BHB, BHBA, and Trolox. The aim of this design was the multifaceted activity of the new compounds, as well as the reduction in their acidic character in order to ensure a safer profile for their use, especially regarding their gastrointestinal toxicity [4]. Machine learning classification, retrieving knowledge from the ChEMBL database, predicted the potential antioxidant activity, strengthening our hypothesis. The newly designed compounds showed an improved overall antioxidant profile in vitro, by a 25-fold improvement in the inhibition of lipid peroxidation, and a 2–10-fold improvement in radical scavenging, compared to the parent molecules. This confirms the fact that the fusion of the used NSAIDs and their corresponding alcohols with the antioxidants BHB, BHBA and Trolox not only leaves their antioxidant profile intact, but further improves it. The overall anti-inflammatory activity was also improved, as shown by both in vitro and in vivo assays. IC_50_ values for LOX-3 inhibition were found to be improved in almost all cases of the new derivatives, reaching values even below 10 μM, whereas the parent compounds had IC_50_ values of above 150 μM (except for compounds **c** and **d**). Molecular docking simulations validated the improved activity and revealed their binding mode at the active site of LOX-3. The improvement (2–4-fold) of the in vivo anti-inflammatory effect, compared to parent NSAIDs (or their corresponding alcohols), was confirmed as evaluated by rat/mouse paw edema reduction. Finally, some selected derivatives which were evaluated in an in vivo hyperlipidemia mouse protocol showed promising results as they reduced total cholesterol and triglyceride levels in plasma, adding to the antihyperlipidemic effect of their parent NSAID [27]. All the aforementioned results contribute to the fact that the designed antioxidant-modified NSAIDs serve their aim of design, that of rendering this approach promising for addressing multifactorial diseases such as atherosclerosis or neurodegeneration, where inflammation and oxidative stress conditions have a synergistic effect.

## Figures and Tables

**Table 1:** Effect of the derivatives on LOX-3, expressed as IC_50_ (μM), after 5 min of incubation.

Compound	IC_50_ Value (μM)	Compound	IC_50_ Value (μM)
**1**	22	**11**	142
**3**	41	**12**	122
**4**	64	**13**	68
**5**	>200 ^a^	**14**	>150 ^a^
**6**	>150 ^a^	**15**	9
**7**	152	**16**	54
**8**	150	**17**	>150 ^a^
**9**	108		
**10**	15		

^a^ Insufficient solubility at higher concentrations, no significant inhibition at that concentration.

**Table 2:** Effect of compounds **1–17** and parent NSAIDs (or corresponding alcohol derivatives) on carrageenan-induced mouse paw edema.

Compound	% Edema Reduction	Compound	% Edema Reduction
**1**	26****	**17**	45**
**2**	56****	**a**	57*** [5]
**3**	78**^a^ [11]	**b**	66****
**4**	49**	**c**	51** [11]
**5**	55****	**d**	40**
**6**	67***	**e**	60*** [5]
**7**	38*	**f**	43****
**8**	48****	**g**	19**^a^ [17]
**9**	63**	**h**	22****
**10**	54****	**i**	35*
**11**	40****	**j**	42*
**12**	54***	**k**	61*** [5]
**13**	39****	**m**	55*** [5]
**14**	42*	BHB	53****
**15**	55****	BHBA	71****
**16**	54***	Trolox	46****

The effect on edema is expressed as a percentage of reduction in edema in comparison to controls. The tested compounds were administered, *i.p.*, at a dose of 0.30 mmol/kg of body weight. Each value represents the mean obtained from 6 animals. ^a^ The tested compound was administered, *i.p.*, at a dose of 0.15 mmol/kg of body weight. Significant difference from carrageenan-treated control: * *p* < 0.01, ** *p* < 0.005, *** *p* < 0.001, **** *p* < 0.0001.

## Data Availability

The data presented in this study are available on request from the corresponding author.

## Supplementary Materials

The following are available online, Figure S1: Confusion Matrix of Support Vector Machine algorithm. Figure S2: Surface mapping of binding pocket and 13(S)-hydroperoxy-9(Z),11(E)-octadecadienoic acid (13S-Hpode) of crystal structure with PDB id 1IK3. Figure S3: Superimposition of crystallographic position of 13S-Hpode (PDB id 1IK3) and EPG (PDB id 1JNQ).

## Sample Availability

Samples of the compounds are available from the authors.
