# Supplementary material for: Design of Multifaceted Antioxidants: Shifting towards Anti-Inflammatory and Antihyperlipidemic Activity
Source: Molecules. 2021 Aug 14;26(16):4928. doi: 10.3390/molecules26164928 (PMC8399014; doi:10.3390/molecules26164928)

Supplementary Materials

Figure S1: Confusion Matrix of Support Vector Machine algorithm. Class 1: LOX-3 inhibitors, Class 2: DPPH inhibitors, Class 3: LOX-3/DPPH decoy set.

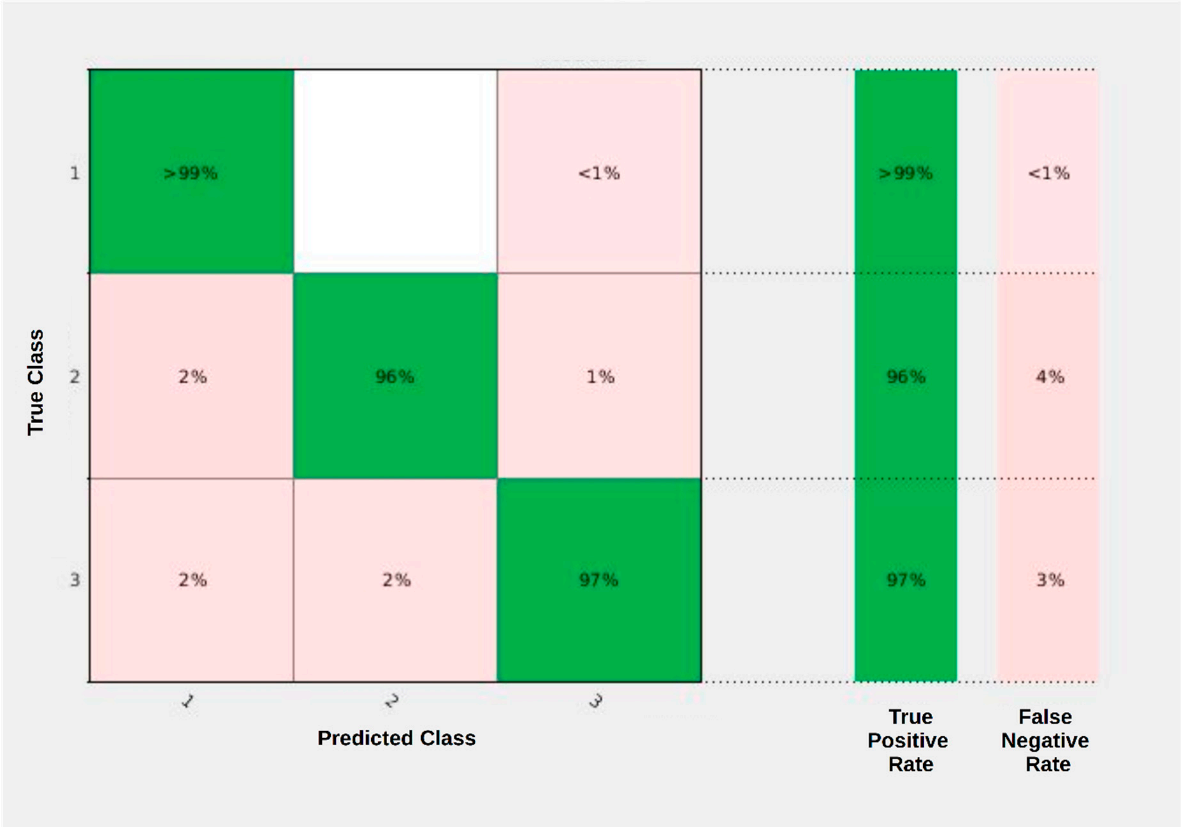

Figure S2: Surface mapping of binding pocket (gray) and 13(S)-hydroperoxy-9(Z),11(E)-octadecadienoic acid (13S-Hpode) (dark blue) of crystal structure with PDB id 1IK3. The binding pocket is shown large enough to accommodate even molecules bulkier to 13S-Hpode. The protein backbone is represented by white ribbons. W519 is pointing the position where 13S-Hpode is bent to adopt an "L" shape.

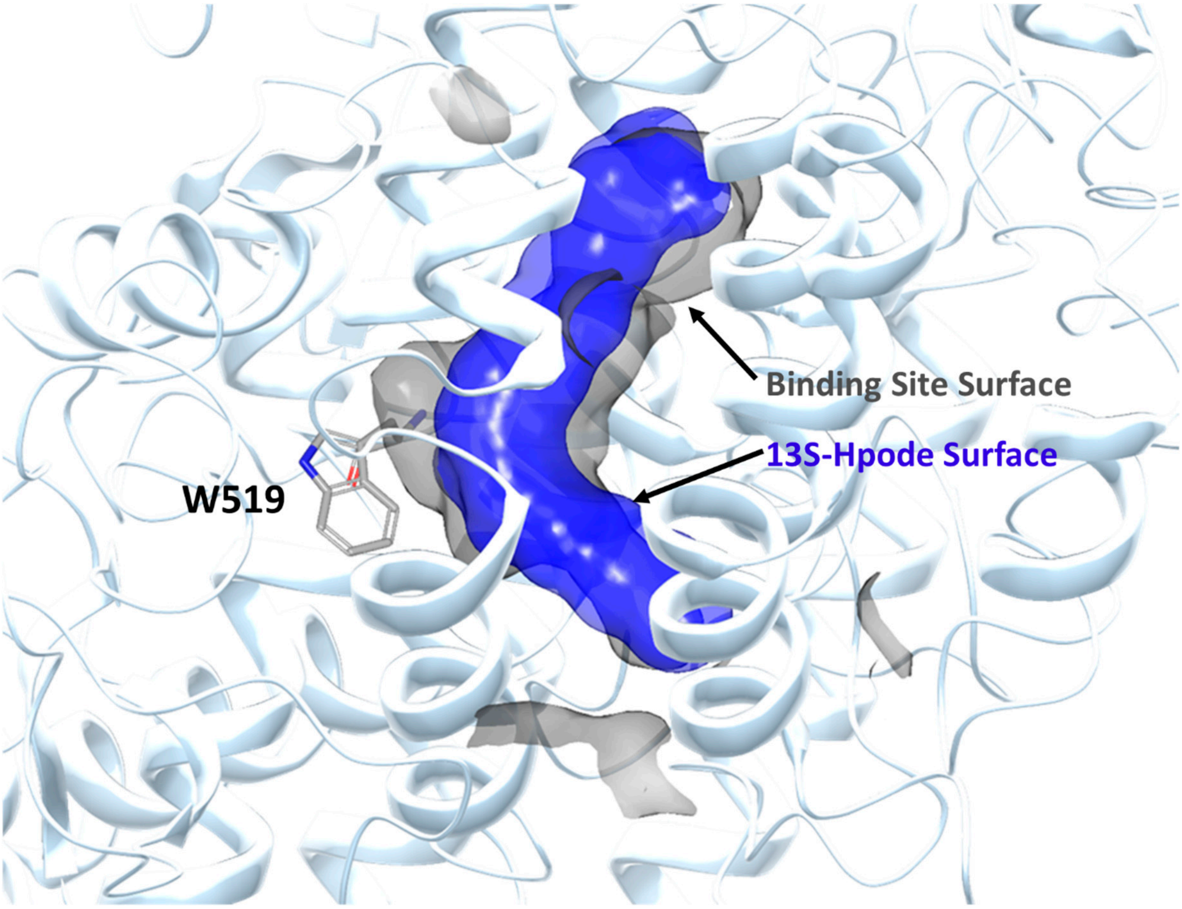

Figure S3: Superimposition of crystallographic position of 13S-Hpode (PDB id 1IK3) (green) and EPG (PDB id 1JNQ) (blue). Both crystallographic ligands are bent next to W519 and their center of mass is positioned at the same coordinates. Both proteins are tightly overlapping and are represented by white ribbons.

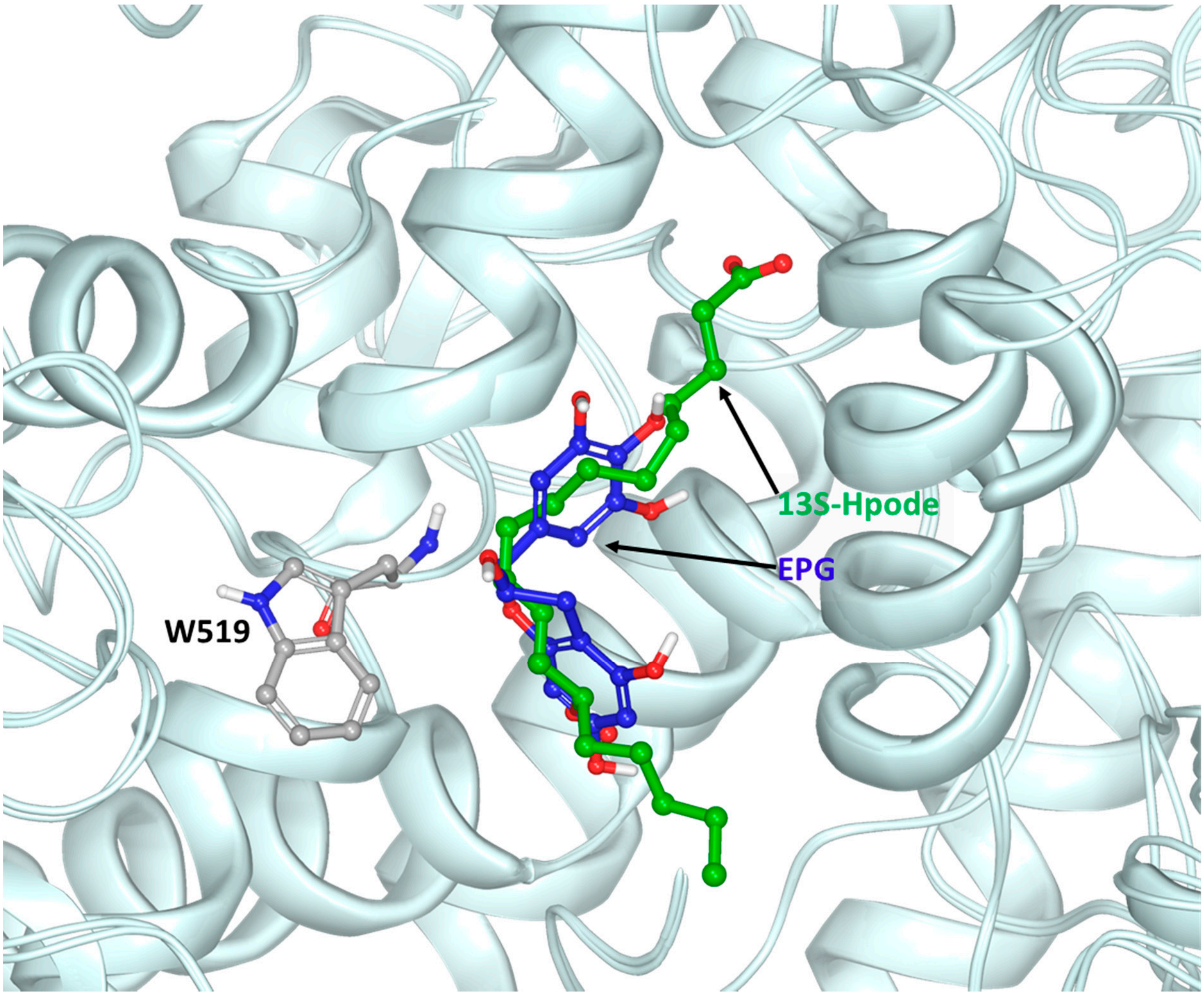

Supplement: Supplementary file 1 [file molecules-26-04928-s001.zip › molecules-1329210-supplementary.pdf]
